# Ceruloplasmin, transferrin and apolipoprotein A-II play important role in treatment's follow-up of paracoccidioidomycosis patients

**DOI:** 10.1371/journal.pone.0206051

**Published:** 2018-10-25

**Authors:** Tatiane Fernanda Sylvestre, Ricardo de Souza Cavalcante, Julhiany de Fátima da Silva, Anamaria Mello Miranda Paniago, Simone Schneider Weber, Bianca Alves Pauletti, Lídia Raquel de Carvalho, Lucilene Delazari dos Santos, Rinaldo Poncio Mendes

**Affiliations:** 1 Universidade Estadual Paulista (UNESP), Faculdade de Medicina de Botucatu, Botucatu, São Paulo–Brazil; 2 Universidade Federal de Mato Grosso do Sul (UFMS), Faculdade de Medicina, Campo Grande—Brazil; 3 Instituto de Ciências Exatas e Tecnologia (ICET), Universidade Federal do Amazonas (UFAM), Itacoatiara—Brazil; 4 Laboratório Nacional de Biociências–CNPEM, Campinas, São Paulo—Brazil; 5 Universidade Estadual Paulista (UNESP), Instituto de Biociência de Botucatu, Botucatu, São Paulo–Brazil; 6 Centro de Estudos de Venenos e Animais Peçonhentos (CEVAP), UNESP, Botucatu, São Paulo—Brazil; University of Texas at San Antonio, UNITED STATES

## Abstract

Paracoccidioidomycosis (PCM) is a systemic disease caused by thermodymorphic fungi of the *Paracoccidioides brasiliensis* complex, (*Paracoccidioides* spp.). Patients with PCM reveal specific cellular immune impairment. Despite the effective treatment, quiescent fungi can lead to relapse, usually late, the serological diagnosis of which has been deficient. The present study was carried out with the objective of investigating a biomarker for the identification of PCM relapse and another molecule behaving as an immunological recovery biomarker; therefore, it may be used as a cure criterion. In the evolutionary analysis of the proteins identified in PCM patients, comparing those that presented with those that did not reveal relapse, 29 proteins were identified. The interactions observed between the proteins, using transferrin and haptoglobin, as the main binding protein, were strong with all the others. Patient follow-up suggests that cerulosplamin may be a marker of relapse and that transferrin and apolipoprotein A-II may contribute to the evaluation of the treatment efficacy and avoiding a premature decision.

## Introduction

Paracoccidioidomycosis (PCM) is a systemic disease caused by thermodymorphic fungi of the *Paracoccidioides brasiliensis* complex, (*Paracoccidioides* spp.). Using molecular taxonomy, the complex is proposed to consist of 5 distinct species, which have been recently described as new taxonomic species. [1–4]

PCM is associated with high morbidity, but low mortality. Since it is not a compulsory reportable disease in Brazil, the actual prevalence of PCM cannot be calculated. A study based on death certificates showed an average annual mortality rate of 1.45 per million inhabitants and was the eighth leading cause of mortality due to predominantly chronic or repetitive disease, infectious and parasitic, and the highest mortality rate among systemic mycoses [5]. The region of Botucatu (São Paulo, Brazil) is considered hyperendemic for PCM [6].

Patients with PCM reveal specific cellular immune impairment [7,8]. Several failures of the cellular immune response were detected in patients with PCM: increased production of TNF-α, activation of the NLRP3 inflammasome, and high counts of CD14^+^CD16^++^ inflammatory monocytes [9]. The immunological alterations observed in patients with the chronic form of the disease during and after treatment may be associated with hypoxia due to pulmonary fibrosis and emphysema. Activation of some transcription factors, such as hypoxia-inducible factors (HIF) [10], may induce growth factor signaling, proinflammatory cytokine release, co-stimulatory molecule expression and lymphocyte proliferation [11, 12].

Upon discontinuation of treatment, patients will depend on the cellular immunity recovered to keep the undissolved fungi quiescent. Therefore, the treatment success depends on whether adequate recovery of cellular immunity has been achieved. The currently available tests for evaluation of cellular immunity are not yet incorporated into a routine clinical laboratory, its costs and its relative complexity. Therefore, a test is still being sought, which may be a biological marker, which will give greater security to the decision to stop treatment.

Despite effective treatment, quiescent fungi may lead to relapse, usually late-about five years after the end of treatment, in both acute / subacute and chronic forms, whose serological diagnosis has been poor [13]. Sylvestre and collaborators observed that the sensitivity of the double immunodiffusion agar gel (DID) reaction was only 45% at relapse and that the enzyme-linked immunosorbent assay (ELISA) was slightly better (65%), but with sensitivity well below what was observed in the same patients on admission.

The low frequency of serological diagnosis of relapse suggests that another disease is manifesting itself, leading to unjustified treatments, further delaying diagnosis and specific therapy, with serious consequences, such as the impairment of previously saved organs, the aggravation of initially and increased sequelae.

In view of the above, the present study was carried out with the objective of investigating a biomarker for the identification of PCM relapse and another molecule behaving as an immunological recovery biomarker; therefore, it may be used as a cure criterion.

## Materials and methods

A retrospective study was performed in patients attending the Clinical Mycology Outpatient Service of the Discipline of Infectious and Parasitic Diseases of the Faculdade de Medicina de Botucatu–São Paulo State University–UNESP. Nine patients with PCM were studied and characterized as either confirmed or probable cases. The need to adhere to treatment was reiterated to all patients at all outpatient visits, and they were also instructed to cease alcohol consumption and smoking.

### Inclusion criteria

#### PCM cases caused by *Paracoccidioides* spp

Confirmed cases were characterized by the presence of a suggestive clinical picture, the identification of typical forms of the yeast phase of the *Paracoccidioides* spp. in one or more clinical tests, and the positive double-immunodiffusion reaction that emerged using an antigen prepared from Pb B-339 [14]. We also included probable cases, characterized by the presence of a suggestive clinical picture and antibodies detected by the double-immunodiffusion reaction, using antigen prepared from Pb B-339. These characterizations were made upon the patient’s admission to the Infectology Service of Botucatu Medical School–UNESP.

### Exclusion criteria

The presence of other systemic diseases of infectious, inflammatory, or neoplastic origin, including co-morbidities, or those resulting from gestation and lactation, were considered as exclusion criteria.

### Classification of clinical forms

The classification of the clinical forms and severity of each patient was made by the infectologist who attended to these patients [15, 16].

### Appropriate treatment and definition of relapse

The treatment was considered adequate when the patient progressed until reaching an apparent cure. A cure could include a clinical cure (the disappearance of signs and symptoms characterizing the disease, as well as normalization of the erythrocyte sedimentation rate [ESR]), as well as a serological (persistent resistance of a double-immunodiffusion reaction for 1 year under consolidated antifungal treatment), radiological (the disappearance of compatible lung lesions upon the activity and onset of residual lesions, such as those associated with fibrosis and emphysema) and, finally, an apparent cure (characterized by the above criteria) maintained for two years after medication suspension [15].

Relapse was characterized by the resurgence of PCM-compatible signs and symptoms in initially confirmed or probable cases associated with the identification of typical forms of the yeast phase of *Paracoccidioides* spp. in any clinical specimen and/or on identification of specific antibodies by double immunodiffusion. It was also characterized by an apparent cure following co-trimoxazole (CMX) treatment. None of the relapsed cases presented any clinical or laboratory evidence of disease of another etiology.

Two types of relapse were considered: a) Clinical and mycological, characterized by the reappearance of signs and symptoms compatible with PCM, and associated with the identification of typical forms of the yeast phase of *Paracoccidioides* spp. in any clinical specimen; and b) clinical, as defined by the reappearance of clinical manifestations compatible with PCM, and which was responsive to antifungal treatment.

### Study groups and research design

Group 1 (G1): five patients from the Botucatu region, with PCM caused by *Paracoccidioides* spp., who presented posterior relapse, evaluated in three different stages: pre-treatment, relapse and post-treatment of relapse;
Group 2 (G2): formed by four patients from the Botucatu region, with PCM caused by *Paracoccidioides* spp., who did not present relapse, evaluated in two different stages: before and after treatment;
Group 3 (G3): consisting of three healthy individuals, living in the Botucatu Region and blood donors at Botucatu Blood Center.

Table 1 presents the characterization of patients according to gender, age, clinical form and diagnosis, and healthy controls according to sex and age.

**Table 1:** Characterization of study groups according to gender, age and clinical form.

Groups	Individual	Gender	Clinical form	Age (years)				Diagnosis	
				to admission	after treatment	at relapse	after treatment of relapse	to admission	at relapse
**Group 1** **Patients with PCM and relapse**	01	M	CF	42	-	61	62	MD / CP sputum	Clinical / MD
	02	M	CF	50	-	61	62	HP oral mucosa	Clinical–cure with CMX*
	03	M	CF	34	-	46	50	CP sputum	Clinical–cure with CMX
	04	F	AF	15	-	19	22	CP lymph node	Clinical / HP lymph node
	05	F	AF	85	-	90	91	CP linfonodo / HP skin	Clinical / HP skin
**Group 2** **Patients with PCM and without relapse**	06	M	CF	54	62	-	-	MD sputum / HP oral mucosa	-
	07	M	CF	55	63	-	-	HP oral mucosa	-
	08	M	AF	38	43	-	-	HP lymph node	-
	09	M	AF	17	23	-	-	MD / HP skin	-
**Group 3** **Healthy individuals**	10	M	-	35	-	-	-	-	-
	11	M	-	29	-	-	-	-	-
	12	F	-	42	-	-	-	-	-

CF- chronic clinical form; AF—acute/subacute clinical form; M- male; F- female; MD- mycological direct; CP sputum—cytopathologic sputum; HP oral mucosa—histopathology of oral mucosa; CP lymph node—cytopathologic node; HP skin—histopathological skin; HP lymph node—histopathology of lymph node and CMX—cotrimoxazole;

*patient presented a strongly positive ELISA test for the diagnosis of PCM relapse.

### Blood collection and serum storage

Patients’ serum samples from the G1, G2 and G3 groups were collected and stored in a freezer at –80°C at the Tropical Diseases Research Laboratory–Medical Mycology, Botucatu Medical School–UNESP.

### Proteomic analysis

Proteomic analysis was performed in four steps: protein quantification, protein digestion in solution, peptide sequencing by mass spectrometry (MS), and data analysis to identify the proteins.

#### Protein quantification

The proteins present in the serum samples were quantitated in triplicate by Bradford’s method [17], with bovine albumin (BSA) as the standard protein.

#### Sample preparation

Serum samples were submitted to enzymatic digestion in solution. To this end, the reduction and alkylation steps were initiated using 10 mM dithiothreitol (DTT) and 45 mM iodoacetamide (IAA), respectively, both of which were solubilized in 50 mM ammonium bicarbonate solution. Then, the samples were submitted to hydrolysis in the presence of the enzyme trypsin at a concentration of 1:50 (enzyme: substrate), solubilized in 50 mM ammonium bicarbonate buffer (pH 7.8), which occurred for 18 hours; it was interrupted with the addition of 1% (v/v) formic acid to the sample volume. The samples were then desalted using Sep-Pak Vac C18 cartridges, reduced in SpeedVac and maintained at 4°C until the time of analysis by MS [18].

#### Peptide sequencing by MS

To identify the most abundant, differentially expressed proteins present in the serum of PCM patients caused by *Paracoccidioides* spp., the samples were submitted to shotgun analysis, where all proteins were hydrolyzed serially and subjected to the spectral counting label-free proteomics analysis in a system liquid chromatography tandem MS (LC-MSMS).

MS analysis, quantified in triplicate, was performed at the National Laboratory of Biosciences (LNBio), located at the National Center for Research in Energy and Materials (CNPEM) in Campinas, São Paulo State, Brazil. An aliquot (4.5 μL, concentration of 50 μg) of digested proteins was injected by an analytic column (C18 1.7 μm BEH 130) (100 μm × 100 mm) RP-UPLC (nanoAcquity UPLC), coupled with nano-electrospray tandem MS on a Q-Tof Premier API mass spectrometer at a flow rate of 600 nL/minute. A trapping column (Symmetry C18; 180 μm × 20 mm) was used for sample desalting at a flow rate of 5 μL/minute over 2 minutes. The gradient was 2%–90% acetonitrile in 0.1% formic acid over 45 minutes. The instrument was operated in MS-positive mode, with data continuum acquisition occurring from m/z 100–2,000 Da at a scan rate of 1 second, and an interscan delay of 0.1 seconds [19].

#### Data analysis for protein identification

The MS files were processed using the Software ProteinLynx Global Service (PLGS, v.2.2.5, Waters), along with the Mascot Server v.2.3.01.0 program (Matrix Science Ltd.). The following parameters were used: cleavage lost by trypsin; the fixed modification of carbamidomethylation; the variable modification of methionine oxidation; 0.1 Da mass tolerance for MS; and 0.1 Da mass tolerance for MSMS. Searches were conducted using the NCBI database (*Homo sapiens* taxonomy, 33,695,097 sequences; available at http://www.ncbi.nlm.nih.gov/protein/?term=homo%20sapiens) containing 92,180 sequences and 36,693,332 residues. For protein quantification, the data were submitted to the Scaffold Q + analysis program (version 3.4.5; Proteome Software, Portland, OR, USA) obtaining normalized spectral count values for each identified protein.

After identification, the proteins were characterized according to the main functions they performed, and the primary proteins in each group were subsequently characterized. The networks of protein interactions against the differentially expressed proteins were analyzed using the STRING 10 tool. The STRING database ("Research Tool for Gene Recovery / Interagent Proteins") represents a continuous effort to analyze protein-protein interactions under a common framework. For associations made using the STRING database, the trust values are assigned to each. How scores are derived by benchmarking the performance of predictions against a common reference set of true and reliable associations [20].

### Statistical analysis

The results were presented as the mean and standard deviation. Single factor analysis of variance and Tukey test was used to compare means for independent samples. Student’s *t*-test was used to compare means for two dependent samples. Statistical tests were performed using the program SAS (Statistical Analysis Software), version 9.4. Significance was set-up at p≤0.05.

For the quantitative evaluation, protein concentrations were defined as either reduced or differentially expressed following comparison with the control group.

### Research ethics committee

The study was approved by the Research Ethics Committee of the Faculty of Medicine of Botucatu—UNESP, number 2,100,907, CAAE submission 21822813.9.0000.5411. Written informed consent for participation was given by the patient or parents.

## Results

### Comparative evaluation of the results observed before treatment (at admission) in groups G1, G2 and G3

There were 29 serum proteins identified in these groups. Eight proteins were categorized as transport, fifteen as immunomodulatory and six others, less abundant, such as activation / regulation of the complement system, activation of the coagulation pathway / protease inhibition, transport / lipid metabolism, protease inhibition / lipid metabolism, protease inhibition and matrix protein.

#### Qualitative analysis

The ceruloplasmin protein was not present in 3 individuals of the PCM relapse patients (Group 1) (Table 2). Alpha-1-antichymotrpsin was absent in the control group (Table 2). The immunoglobulin (Ig) alpha-2 chain C region was not detected in any of the groups (Table 2). Complement factor B was not present in the group of patients with PCM with relapse (Table 2). Alpha globin was not present in 3 individual of the PCM relapse patients (Group 1) and control group (Group 3) (Table 2).

**Table 2:** Qualitative analysis of serum proteins of patients with paracoccidioidomycosis—G1 and G2 groups and healthy subjects—G3 group, before treatment (at admission).

Protein	Access code	[G1] PCM with relapse		[G2] PCM without relapse		[G3] Control group (n = 3)
		AF (n = 1)	CF (n = 2)	AF (n = 2)	CF (n = 2)	
**1.** *Serum albumin*	P02768.2	+	+	+	+	+
**2.** *Transferrin*	P02787.3	+	+	+	+	+
**3.** *Apoliprotein A-I*	P02647.1	+	+	+	+	+
**4.** *Haptoglobin*	P00738.1	+	+	+	+	+
**5.** *Ig kappa chain C region*	P01834.2	+	+	+	+	+
**6.** *Ig gamma-1 chain C region*	P01857.1	+	+	+	+	+
**7.** *Ig lambda-2 chain C region*	P0CG05.1	+	+	+	+	+
**8.** *Alpha-2-macroglobulin*	P01023.3	+	+	+	+	+
**9.** *Ig alpha-1 chain C region*	P01876.2	+	+	+	+	+
**10.** *Alpha-1-antitrypsin*	P01009.3	+	+	+	+	+
**11.** *Hemopexin*	P02790.2	+	+	+	+	+
**12.** *Ig gamma-2 chain C region*	P01859.2	+	+	+	+	+
**13.** *Alpha-1-acid-glycoprotein*	P02763.1	+	+	+	+	+
**14**. *Complement C3*	P01024.2	+	+	+	+	+
**15.** *Apolipoprotein A-II*	P02652.1	+	+	+	+	+
**16.** *Ig gamma-3 chain C region*	P01860.2	+	+	+	+	+
**17.** *Ig gamma-4 chain C region*	P01861.1	+	+	+	+	+
**18.** *Vitamin D-Binding Protein*	P02774.1	+	+	+	+	+
**19.** *Ceruloplasmin*	P00450.1	-	-	+	+	+
**20.** *Complement C4-A*	P0C0L4.2	+	+	+	+	+
**21.** *Alpha-1-antichymotrypsin*	P01011.2	+	+	+	+	-
**22.** *Kininogen*	P01042.2	+	+	+	+	+
**23.** *Ig alpha-2 chain C region*	P01877.3	-	-	-	-	-
**24**. *Beta-globin*	P68871.2	+	+	+	+	+
**25.** *Ig kappa chain V-III*	P04433.1	+	+	+	+	+
**26.** *Beta-2-glycoprotein 1*	P02749.3	+	+	+	+	+
**27.** *Ig heavy chain V-III TIL*	P01764.2	+	+	+	+	+
**28.** *Complement factor B*	P00751.2	-	-	+	+	+
**29.** *Alpha-globin*	P69905.2	-	-	+	+	-

+ present;—absent; AF—acute / subacute clinical; CF- chronic clinical form and n- number of participants.

#### Quantitative analysis

**Fig 1:**
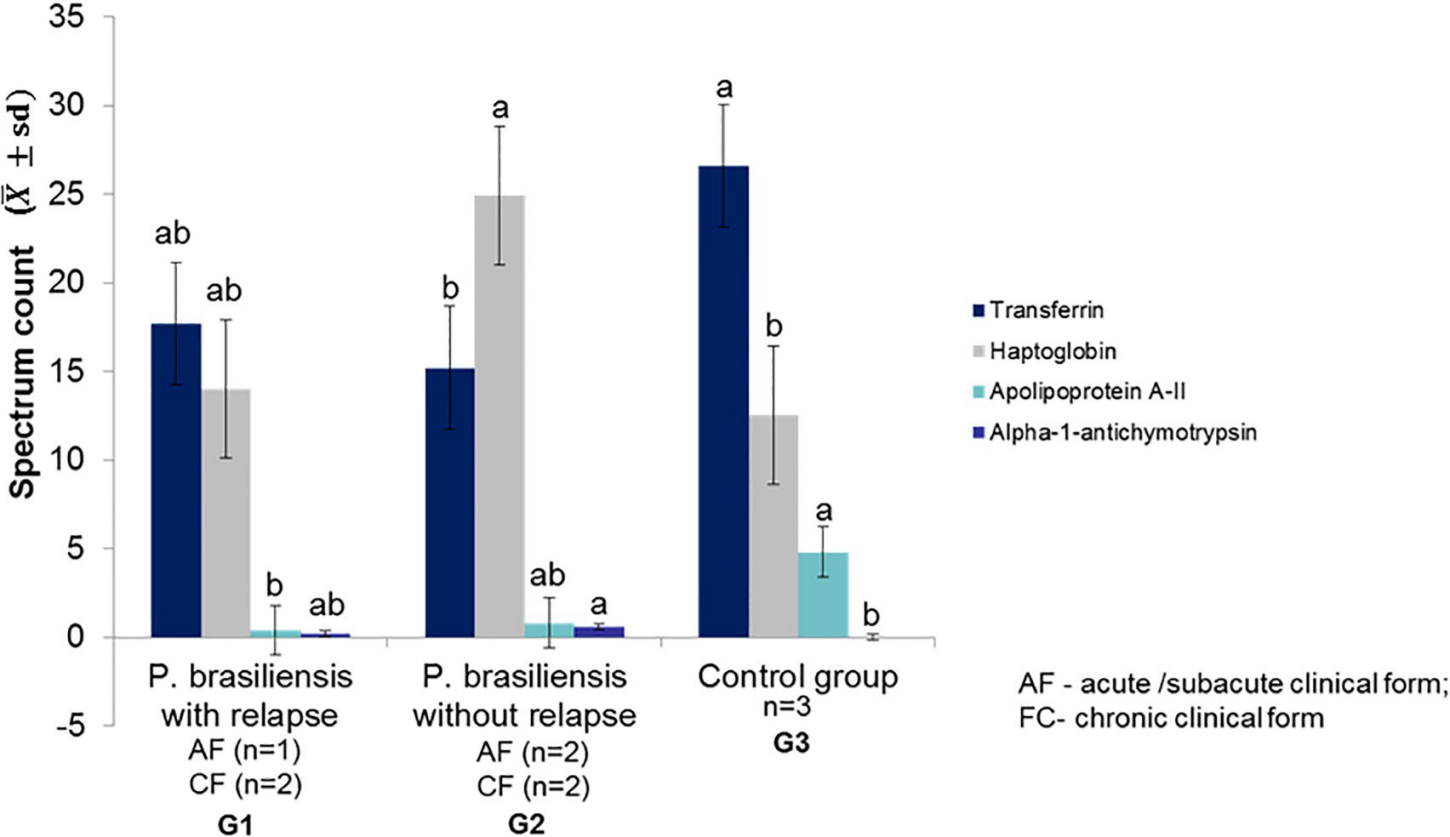
Protein serum levels presented differences among groups, at admission. Small letters compare every protein values among groups (G1 *vs*. G2 *vs*. G3); Small letters compare every protein values among groups (G1 *vs*. G2 *vs*. G3); means followed by the same letter are not different (p>0.05), while means followed by different letters show significant differences (p≤0.05); Group 2: patients with paracoccidioidomycosis and without relapse; Group 3: healthy individuals. Statistical analysis: analysis of variance and Tukey test.

The protein dosages are found in the supporting information S1A, S1B and S1C Table. Transferrin was lower in the samples of G1 & G2 than in the control group (Fig 1).

Haptoglobin and alpha-1-antichymotrypsin were higher in the G2 than in the control group (Fig 1). Apolipoprotein A-II was higher in the control group (Fig 1). The proteins identified did not differ according to clinical form.

#### Network of interactions

**Fig 2:**
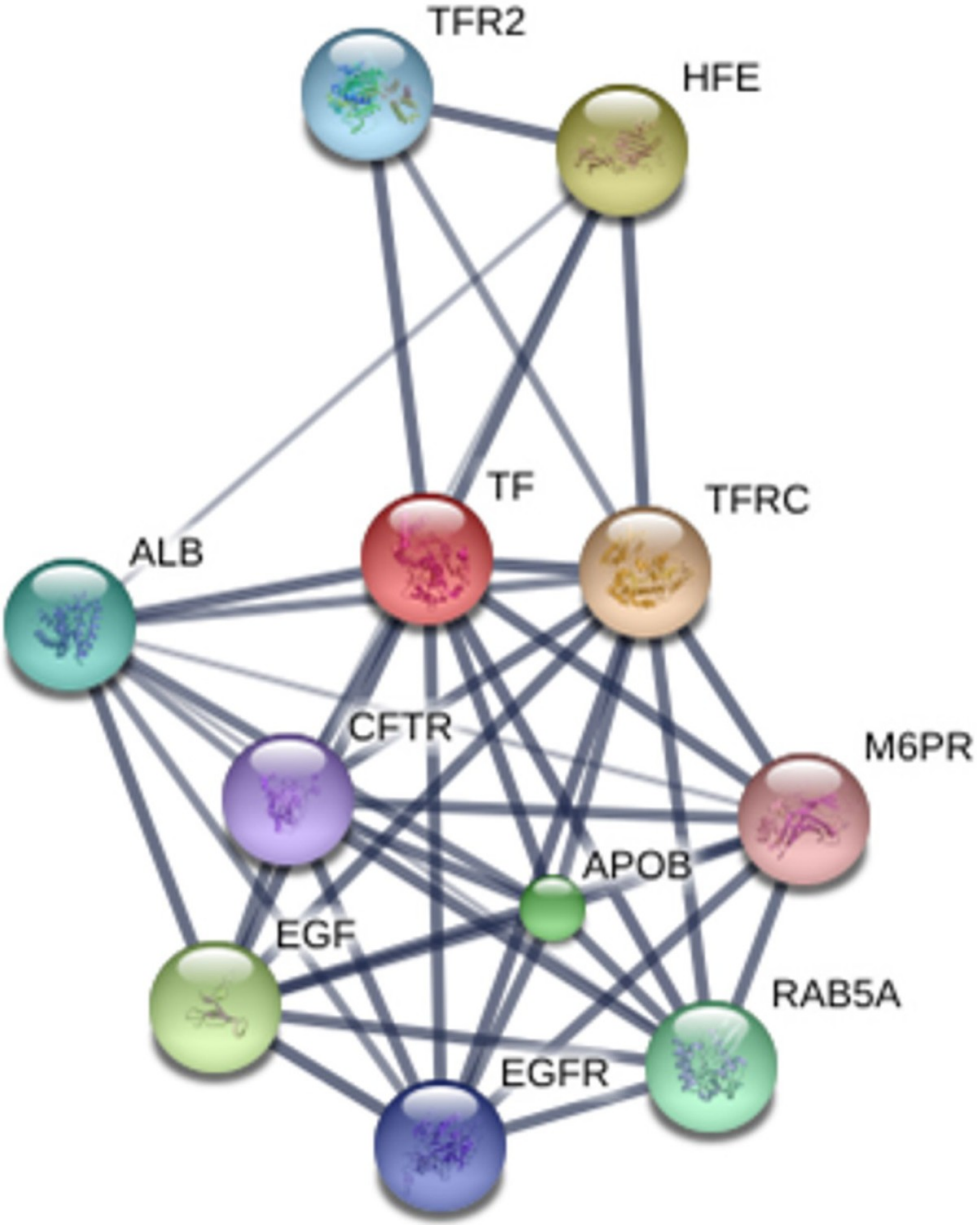
Network of interactions between proteins. TF–*transferrin*; TFRC–*transferrin receptor*; HFE—h*emochromatosis*; EGF—*epidermal growth factor*; APOB—a*polipoprotein B*; RAB5A - *member RAS oncogene family*; ALB—*serum albumin*; TFR2—*transferrin receptor 2*; EGFR—*epidermal growth factor receptor*; CFTR—*cystic fibrosis transmembrane conductance regulator (ATP-binding cassette sub-family C*, *member 7)* and M6PR—*mannose-6-phosphate receptor (cation dependent)*.

**Fig 3:**
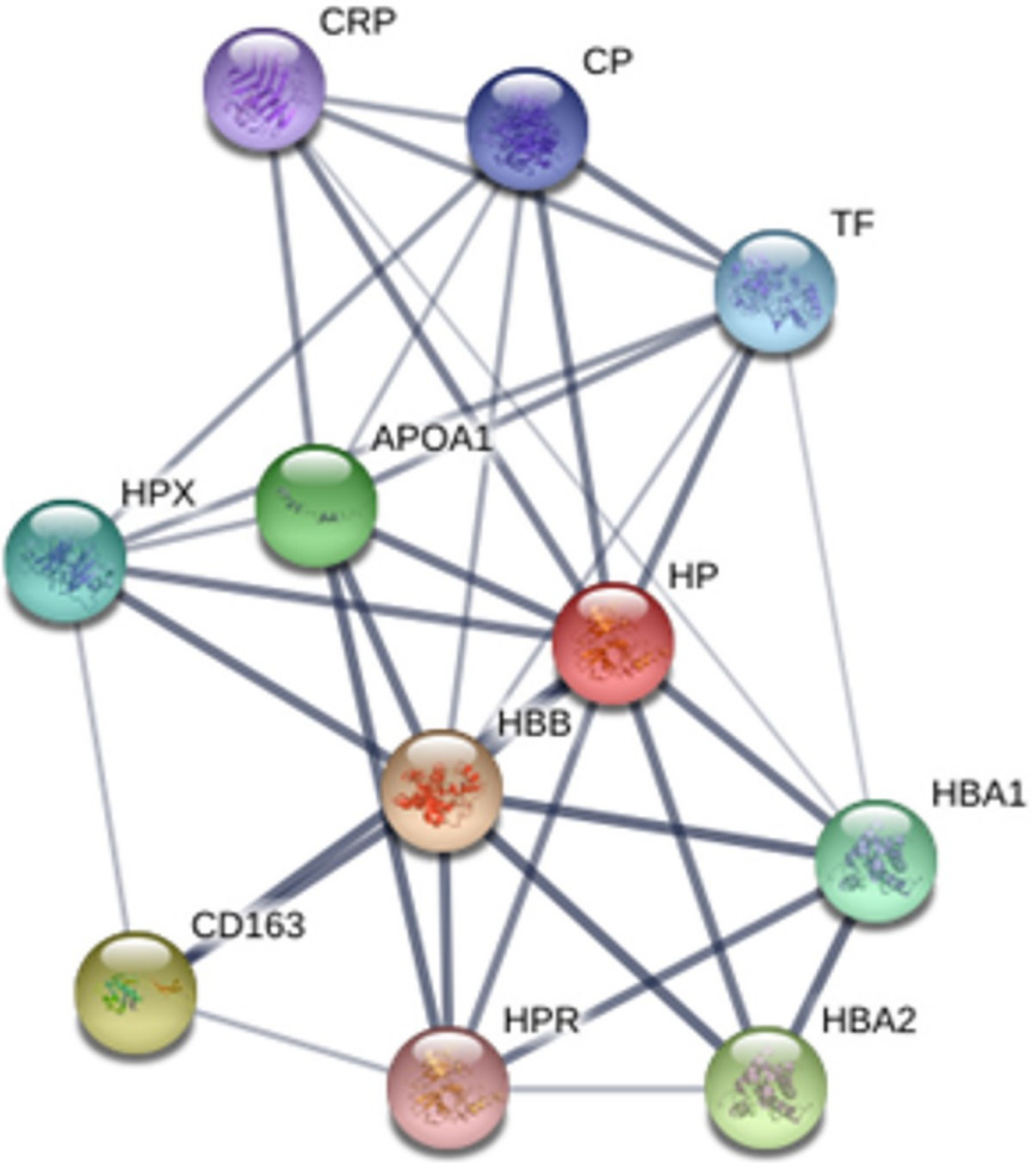
Network of interactions between proteins. HP–*haptoglobin;* HBB—*hemoglobin*, *beta*; CD163—CD163 *molecule*; HBA2—*Hemoglobin*, *alpha 2*; APOA1—*apolipoprotein A-I*; HBA1—*hemoglobin*, *alpha 1*; HPX—*hemopexin*; TF—*transferrin*; CP–*ceruloplasmin*; CRP—*C-reactive protein* and HPR—*haptoglobin-related protein*.

The interactions observed between the proteins, using transferrin and haptoglobin as the major binding proteins, were strong with all the others (Figs 2 and 3).

In our analysis, we used transferrin as the main interaction protein and we found highest connections with all the others (score up to 0.900). Among the main interactions we can highlight the interaction with its receptors (TFFR and TFR2), interaction with HFE (Binds to TFR and reduces its affinity for iron-loaded transferrin) and interaction with ALB (the main protein of serum, and its main function is the regulation of the colloidal osmotic pressure of blood, also its major zinc transporter in serum). Other interactions occur with EGF (epidermal growth factor) and its respective receptor (EGFR), CFTR (ATP-binding cassette sub-family C, member 7), M6PR (mannose-6-phosphate receptor, cation dependent), RAB5A (member RAS oncogene family) and APOB (apolipoprotein B).

### Follow-up of the serum proteins in the relapsed PCM patients (Group G1)–at admission, at relapse and after treatment of relapse

Twenty-two serum proteins were identified in this group. Six proteins were categorized as transport, eleven as immunomodulatory and another five, less abundant, such as activation / regulation of the complement system, activation of the protease inhibition / coagulation pathway and inhibition of protease / lipid metabolism.

#### Qualitative analysis

The Ig gamma-4 chain C region and alpha-1-antichymotrypsin were present in two stages of the active PCM at admission and relapse, being negative after relapse treatment. Ceruloplasmin revealed different behavior at the two stages when PCM was in activity: absent at admission and present at relapse, becoming negative after treatment of relapse (Table 3).

**Table 3:** Qualitative analysis of serum proteins in the group of paracoccidioidomycosis patients with relapse of the disease (Group G1), before treatment (on admission), in relapse and after relapse treatment.

Protein	Access code	Before treatment (n = 3)	At relapse (n = 5)	After the treatment of relapse (n = 5)
**1.** *Serum albumin*	P02768.2	+	+	+
**2.** *Transferrin*	P02787.3	+	+	+
**3.** *Apoliprotein A-I*	P02647.1	+	+	+
**4.** *Haptoglobin*	P00738.1	+	+	+
**5.** *Ig kappa chain C region*	P01834.2	+	+	+
**6.** *Ig gamma-1 chain C region*	P01857.1	+	+	+
**7.** *Ig lambda-2 chain C region*	P0CG05.1	+	+	+
**8.** *Alpha-2-macroglobulin*	P01023.3	+	+	+
**9.** *Ig alpha-1 chain C region*	P01876.2	+	+	+
**10.** *Alpha-1-antitrypsin*	P01009.3	+	+	+
**11.** *Hemopexin*	P02790.2	+	+	+
**12.** *Ig gamma-2 chain C region*	P01859.2	+	+	+
**13.** *Alpha-1-acid-glycoprotein*	P02763.1	+	+	+
**14.** *Complement C3*	P01024.2	+	+	+
**15.** *Apolipoprotein A-II*	P02652.1	+	+	+
**16.** *Ig gamma-3 chain C region*	P01860.2	+	+	+
**17.** *Ig gamma-4 chain C region*	P01861.1	+	+	-
**18.** *Vitamin D-Binding Protein*	P02774.1	+	+	+
**19.** *Ceruloplasmin*	P00450.1	-	+	-
**20.** *Complement C4-A*	P0C0L4.2	+	+	+
**21.** *Alpha-1-antichymotrypsin*	P01011.2	+	+	-
**22.** *Kininogen*	P01042.2	+	+	+

+ present;—absent and n- number of participants.

#### Quantitative analysis

**Fig 4:**
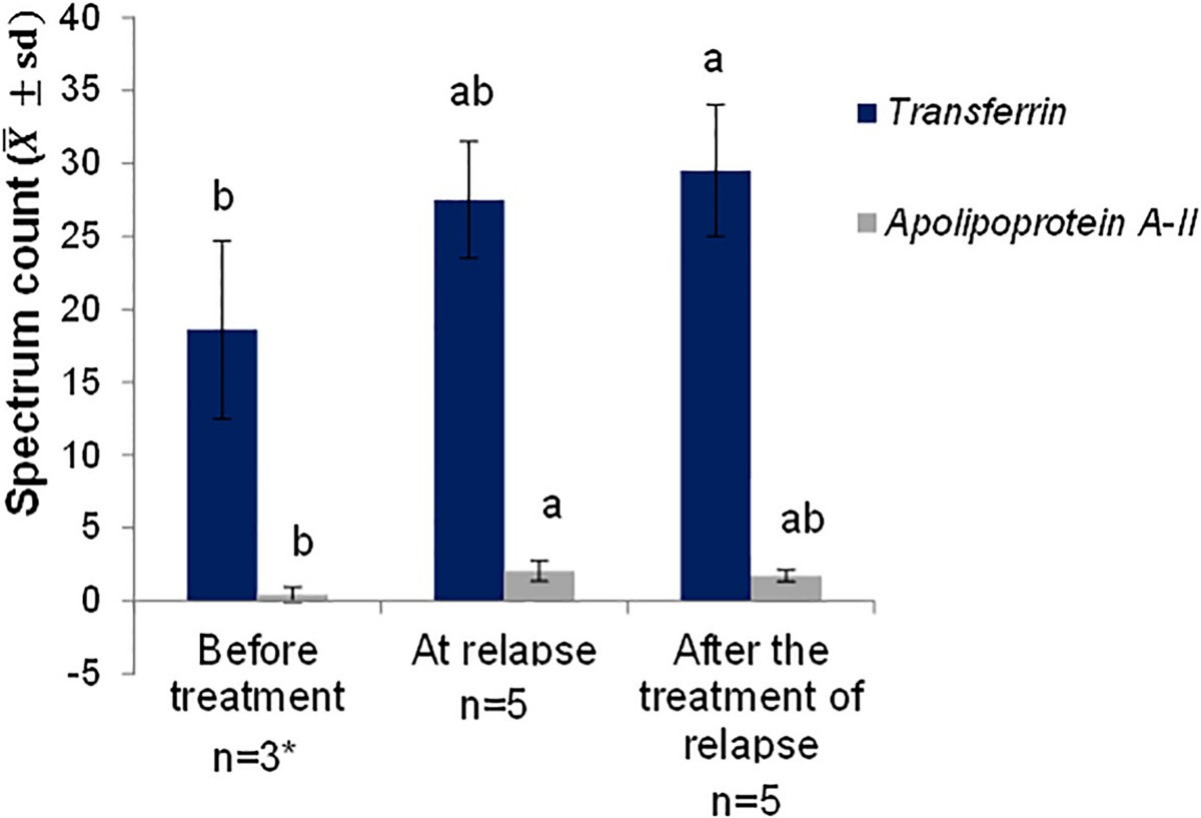
Quantitative evolutionary analysis of serum proteins, presented as mean and standard deviation in five patients with paracoccidioidomycosis and with disease relapse (Group G1), before treatment (at admission), at the time of relapse and after of the treatment of relapse. Small letters compare every protein values among stages (before treatment vs. at the time of relapse vs. after of the treatment of relapse); means followed by the same letter are not different (p>0.05), while means followed by different letters show significant differences (p≤0.05); n- number of participants; * 2 of the 5 samples were not read in the mass spectrometer; group 1: patients with paracoccidioidomycosis and relapse. Statistical analysis: analysis of variance and Tukey test.

The protein dosages are found in the supporting information S2A and S2B Table. Transferrin increased from admission to the post-treatment time of relapse (Fig 4). Apolipoprotein A-II increased from admission to relapse, remaining unchanged after relapse treatment (Fig 4).

#### Network of interactions

**Fig 5:**
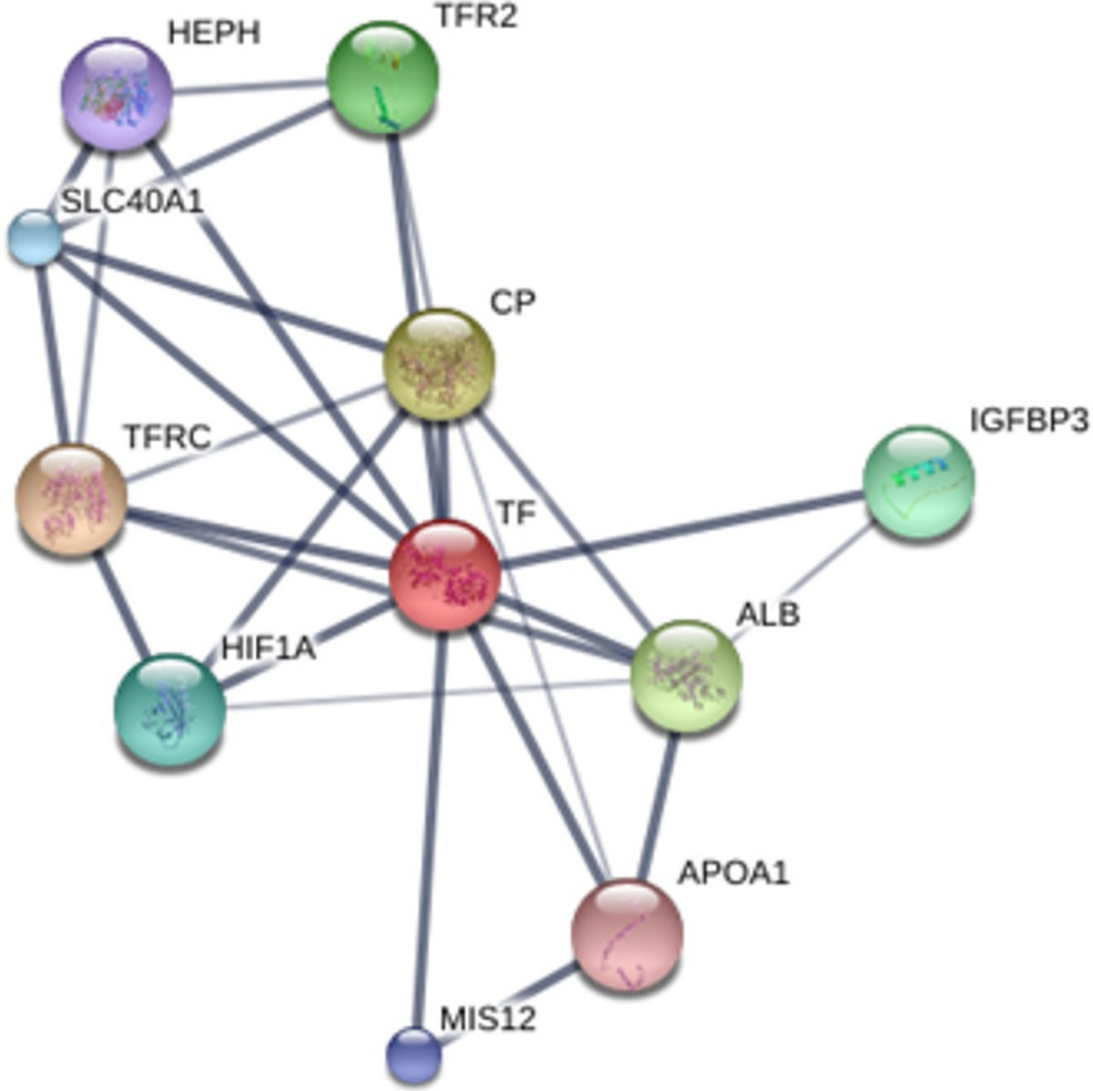
Network of interactions between proteins. TF–*transferrin*; TFRC–*transferrin receptor*; CP—*ceruloplasmin*; ALB–*albumin*; TFR2: *transferrin receptor 2*; IGFBP3 –*insulin-like growth fator binding protein*; HIF1A –*hypoxia inducible fator 1*; SLC40A1 –*solute carrier faily 40 (iron-regulated transporter)*; MIS12 –MIND *kinetochore complex component;* HEPH—*hephaestin* and APOA1—*apoliprotein A-I*.

The interactions observed between the proteins, using transferrin as the major binding protein, were strong with all others (Fig 5).

In this analysis, we used transferrin as the main interaction protein and we found strong connections with all the others. Among the main interactions we can highlight the interaction with it receptor TFRC, interaction with HIF1A (hypoxia inducible factor 1), ALB (the main protein of serum, and its main function is the regulation of the colloidal osmotic pressure of blood, also its major zinc transporter in serum), MIS12 (kinetochore complex component), APOA1 (apolipoprotein A1), CP (ceruloplasmin) and IGFBP3 (insulin-like growth factor binding protein). Other interactions occur with SLC40A1 (iron-regulated transporter, TRF2 (transferrin receptor 2) and HEPH (hephaestin).

### Follow-up of the serum proteins in PCM patients, who did not present relapse (Group G2), before and after treatment

Twenty-two serum proteins were identified in this group. Six proteins were categorized as transport, eleven as immunomodulatory and another five, less abundant, such as complement activation / regulation, protease inhibition / coagulation pathway / transport, lipid transport / metabolism, and protease inhibition / lipid metabolism.

#### Qualitative analysis

The vitamin D-binding, ceruloplasmin, complement C4-A and alpha-1-antichymotrypsin proteins, present at admission, were not identified after treatment (Table 4).

**Table 4:** Qualitative analysis of serum proteins in the group of paracoccidioidomycosis patients (Group G2) before and after treatment.

		Treatment	
Protein	Access code	Before (n = 3)	After (n = 5)
**1.** *Serum albumin*	P02768.2	+	+
**2.** *Transferrin*	P02787.3	+	+
**3.** *Apoliprotein A-I*	P02647.1	+	+
**4.** *Haptoglobin*	P00738.1	+	+
**5.** *Ig kappa chain C region*	P01834.2	+	+
**6.** *Ig gamma-1 chain C region*	P01857.1	+	+
**7.** *Ig lambda-2 chain C region*	P0CG05.1	+	+
**8.** *Alpha-2-macroglobulin*	P01023.3	+	+
**9.** *Ig alpha-1 chain C region*	P01876.2	+	+
**10.** *Alpha-1-antitrypsin*	P01009.3	+	+
**11.** *Hemopexin*	P02790.2	+	+
**12.** *Ig gamma-2 chain C region*	P01859.2	+	+
**13.** *Alpha-1-acid-glycoprotein*	P02763.1	+	+
**14.** *Complement C3*	P01024.2	+	+
**15.** *Apolipoprotein A-II*	P02652.1	+	+
**16.** *Ig gamma-3 chain C region*	P01860.2	+	+
**17.** *Ig gamma-4 chain C region*	P01861.1	+	+
**18.** *Vitamin D-Binding Protein*	P02774.1	+	-
**19.** *Ceruloplasmin*	P00450.1	+	-
**20.** *Complement C4-A*	P0C0L4.2	+	-
**21.** *Alpha-1-antichymotrypsin*	P01011.2	+	-
**22.** *Kininogen*	P01042.2	+	+

+ present;—absent and n- number of participants.

#### Quantitative analysis

**Fig 6:**
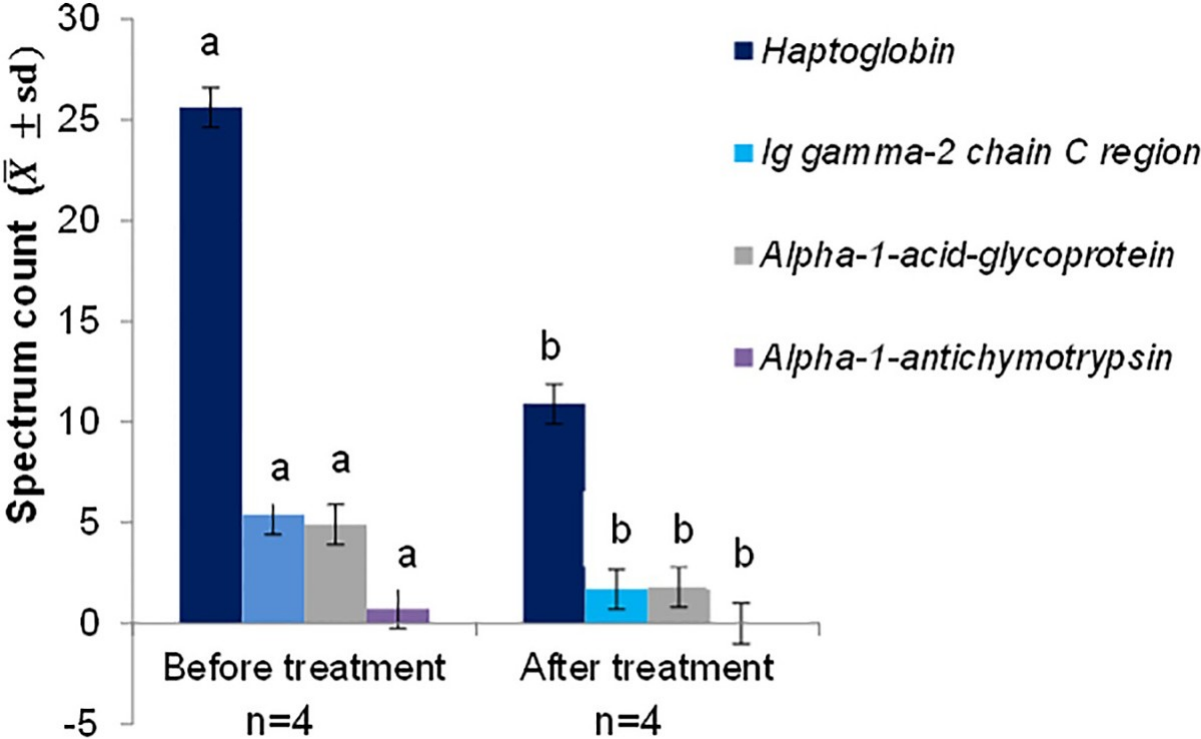
Quantitative evolutionary analysis of serum proteins, presented as mean and standard deviation detected in four patients with paracoccidioidomycosis (Group G2) before and after treatment. Small letters compare every protein values among stages (before vs. after treatment); means followed by the same letter are not different (p>0.05), while means followed by different letters show significant differences (p≤0.05); group 2: patients with paracoccidioidomycosis and without relapse; and n- number of participants. Statistical analysis: Student’s *t*-test.

The protein dosages are found in the supporting information S3A and S3B Table. Haptoglobin, Ig gamma-2 chain C region, alpha-1-acid-glycoprotein and alpha-1-antichymotrypsin were decreased after treatment (Fig 6).

#### Network of interactions

he interactions observed between the proteins, using haptoglobin as the major binding protein, were strong with all the others (Fig 3).

In this analysis, we used haptoglobin as the main interaction protein and we found strong connections with all the others. Among the main interactions we can highlight the interaction with HBB (hemoglobin, beta), HPX (hemopexin), APOA1 (apolipoprotein A1), CD163 molecule, HPR (haptoglobin-related protein.), HBA 1 and HBA2 (hemoglobin, alpha). Other interactions occur with TF (transferrin), CP (ceruloplasmin) and CRP (C-reactive protein).

### Evaluation of serum proteins as a whole

In the evolutionary analysis of the proteins identified in patients with PCM and that did not present relapse, it was observed that the vitamin D-Binding, ceruloplasmin, complement C4-A and alpha-1-antichymotrypsin proteins present at admission were not identified after treatment. In the quantitative analysis, haptoglobin, Ig gamma-2 chain C region, alpha-1-acid-glycoprotein and alpha-1-antichymotrypsin decreased after treatment.

The evolutionary study of the proteins identified in PCM patients and those that relapsed showed that the Ig gamma-4 chain C region and alpha-1-antichymotrypsin were present in the two PCM was active at admission and relapse, and was negative after relapse treatment. Ceruloplasmin revealed different behavior in the two stages when PCM was active: absent at admission and present at relapse, becoming negative after relapse treatment. In quantitative analysis, transferrin increased from admission to post-treatment relapse. Apolipoprotein A-II increased from admission to relapse, remaining unchanged after relapse treatment.

## Discussion

Traditional shotgun proteomics has been used to detect a vast array of proteins through mass spectrometric analysis; it has been considered the standard approach in research to profile protein content in a biological sample which could lead to the discovery of new protein candidates with diagnostic, prognostic, and therapeutic values. The field of clinical proteomics has aroused great interest in the search for protein or peptidic biomarkers, since mass spectrometry has been able to characterize a large number of proteins and their post-translational modifications under different biological conditions. [21–23].

The proteomic methodology has been used for the identification of many agents of infectious diseases, such as bacteria [24,25], protozoa [26,27] and fungi [28–31]. Some proteomic studies have been performed to identify *Paracoccidioides* spp. isolates regarding its species [11–14]. All these studies focused protein from the etiological agents, and none of them analyzed protein from serum samples of the patients.

Pitarch et al [28] isolated and identified 85 proteins from *Candida albicans* isolates using two-dimensional electrophoresis and mass spectrometry. These substances were submitted to immunoblotting assay with sera from four patients with systemic candidiasis, showing 26 different enzymes with antigenic properties. The authors suggest that these proteins could be useful for diagnosing and monitoring the treatment.

Using the same methodology, Martins et al [29] identified 48 proteins and 374 epitopes with antigenic properties, and positive results at immunoblotting assay, using serum from three patients.

Brandão et al [31] determined by immunoproteomics approach the immunoreactivity of synthetic peptides selected from proteins derived from *C*. *gattii*. Six out of 63 B-cell epitopes from previously identified *Cryptococcus gattii* immunoreactive proteins were synthesized and evaluated as antigens in ELISA, were recognized by antibodies in immunoassays, with a specificity of 100%, sensitivity of 78% and low cross-reactivity.

Virginio et al [30] identified 40 antigenic proteins from *Aspergillus fumigatus* which could be useful for early diagnosis using two-dimensional electrophoresis and mass spectrometry (MS / MS). A BLAST analysis revealed that cytochrome P450 and eEF-3 proteins had low homology to human host proteins or etiologic agents of other invasive fungal infections. This is the first study describing antigenic proteins specific for *Aspergillus fumigatus* germination, recognized by the serum of patients with confirmed invasive aspergillosis.

PCM is a systemic granulomatous disease that, in spite of an appropriate treatment, can present late relapse due to the persistence of latent fungi. Bearing this in mind, there are three problems to be considered: 1) What would be the best marker to ensure that the discontinuation of the treatment was not so early? 2) When a treated PCM patient presents clinical manifestations compatible with this mycosis, how to differentiate PCM relapse from other disease, such as acute lymphoma, leukemia, toxoplasmosis, visceral leishmaniasis, and infectious mononucleosis for acute PCM or histoplasmosis, tuberculosis, cutaneous or mucosal leishmaniasis, chromoblastomycosis, sarcoidosis, pulmonary cancer and pneumoconiosis for chronic PCM [13,16,32]? A misdiagnosis could worsen the patient’s condition with serious consequences. 3) At admission, is it possible to detect a biomarker for a late relapse? In case of confirmation that the new clinical manifestations are caused by *Paracoccidioides* spp., how to differ relapse from a new infection?

The serological diagnosis of relapse showed a sensitivity of 45% with the double agar gel immunodiffusion test (DID) and 65% when the enzyme-linked immunosorbent assay (ELISA) was performed; despite this little improvement, these results are much lower than those observed at admission (80%) [13].

Relapse is defined in this study as the reappearance of symptomatology in PCM patients with mycologically and/or serologically (DID) positive results at admission and cured after appropriate treatment, regarding Mendes’ criteria of cure [16]. The relapse was confirmed by mycological and/or serological examinations. However, it is impossible to deny a new paracoccidioidal infection, and the term relapse was used as a clinical approach. Molecular identification should be performed to distinguish between reactivation of latent foci and a new infection [33].

Human plasma contains a large number of proteins, classified as to their functions as transport, immunomodulators, activation and regulation of the complement system, activation of the coagulation pathway, protease inhibition, lipid metabolism and matrix protein. Several proteins perform more than one of these functions. These proteins, as well as tissue molecules, can be used for diagnosing and monitoring the therapy [34]. Although 1,175 proteins have been described in human plasma [35], only 10 of them constitute 95% of the total protein content [36,37]: albumin (54%), immunoglobulin G (17%), alpha-1-antitrypsin (3.8%), alpha-2-macroglobulin (3.6%), immunoglobulin A (3.5%), transferrin (3.3%), haptoglobin (3%), apolipoprotein A-1 (3%), immunoglobulin M (2%) and alpha-1 acid glycoprotein (1.3%).

In the follow-up study of the proteins identified in patients with relapse of PCM it was observed in the qualitative analysis that ceruloplasmin showed different behavior in the two stages in which the PCM was in activity: absent at admission, present at relapse and absent again after treatment of relapse. The presence of ceruloplasmin may be a marker of relapse of PCM, since it was found only at relapse stage. Longo et al. identified the ceruloplasmin bound to the cell wall of *P*. *brasiliensis* [38]. Ceruloplasmin is responsible for transporting 70% of the total copper in human serum and exhibits a copper-dependent oxidase activity, which possibly oxidizes Fe^2+^ to Fe^3+^, thereby participating in iron transport. In addition, the survival and development of *P*. *brasiliensis* and *P*. *lutzii* depend on iron molecules [39].

There is a relationship between hemoglobin, transferrin, ceruloplasmin and transport of iron and oxygen. Most of the iron found in the human body is in the form of hemoglobin in red blood cells. Therefore, phagocytosis and the degradation of red blood cells, made by macrophages, is an important mechanism in the recycling of iron. The macrophage, after phagocytosis, initiates a series of chemical reactions, generating carbon monoxide (CO), iron and bilirubin. Released ferrous iron may be stored as ferritin in the phagocyte or taken to the extracellular medium by ferroportin. Extracellular iron goes into the serum, where it is converted by ceruloplasmin into ferric iron to be able to bind to transferrin, be transported and reach the tissues. Most of the iron present in transferrin comes from recycling and only small amount comes from the diet. The amount of iron varies between the various parts of the body because there are different needs and storage capacities for each tissue. The red blood cells require large amounts of iron to perform the efficient transport of gases while some enzymes often need only one iron atom to stabilize and perform their function. However, serious dysfunctions can be generated if the distribution of iron in the body is not in balance, caused by excess or lack, demonstrating the importance of intracellular and extracellular systemic regulatory mechanisms [39].

Vitamin D-Binding, ceruloplasmin, complement C4-A and alpha-1-antichymotrypsin are proteins present in the inflammatory processes. The follow-up analysis of the proteins identified in patients with PCM that did not present relapse revealed that these proteins, present at admission, were not identified after effective treatment, by the resolution of the inflammatory process of infectious etiology. Quantitative analysis showed a reduction in the levels of haptoglobin, Ig gamma-2 chain C region, alpha-1-acid-glycoprotein and alpha-1-antichymotrypsin after treatment for the same reason previously described.

Ig gamma-4 chain C region and alpha-1-antichymotrypsin, acute phase proteins with immunomodulatory properties, were active at admission and relapse, and negative following treatment of relapse, as expected.

The proteomic assay was used to evaluate more than 20 proteins in the same analysis, aiming to identify those that could indicate a future relapse, when evaluated at admission and those useful as a criterion of cure. It is well known that this methodology could not be standardized in the clinical laboratories. Thus, the next step of this research line is the evaluation of the selected proteins using commercial kits. The study is a preliminary evaluation for potential biomarkers and that further studies are needed to validate their efficacy to serve as biomarkers.

To the best of our knowledge, this is the first time the proteomic methodology was performed in serum samples to identify biomarkers for infectious diseases. Our results suggest that cerulosplamin may be a marker of relapse and that transferrin and apolipoprotein A-II may contribute to the evaluation of cure and discontinuation of treatment, avoiding a premature decision.

## Supporting information

S1 Table **A-C. Serum protein quantification as spectral count, presented as mean and standard deviation in patients with paracoccidioidomycosis—G1 and G2 groups and healthy subjects—G3 group, before treatment (at admission).** Means with the same letters in bold do not differ statistically from each other, whole means with different letters do differ (p≤0.05); AF—acute / subacute clinical form; CF- chronic clinical form; n- number of participants and … sequence not in database; Group 1: patients with paracoccidioidomycosis and relapse; Group 2: patients with paracoccidioidomycosis and without relapse; Group 3: healthy individuals. Statistical analysis: analysis of variance and Tukey test.
(DOCX)

S2 Table **A-B. Serum protein quantification as spectral count, presented as mean and standard deviation in five patients with paracoccidioidomycosis and with disease relapse (Group G1), before treatment (at admission), at the time of relapse and after treatment of relapse.** Means with the same letters in bold do not differ statistically from each other, whole means with different letters do differ (p≤0.05); n- number of participants and group 1: patients with paracoccidioidomycosis and relapse. Statistical analysis: analysis of variance and Tukey test.
(DOCX)

S3 Table **A-B. Serum protein quantification as spectral count, presented as mean and standard deviation in four patients with paracoccidioidomycosis (Group G2) before and after treatment.** Means with the same letters in bold do not differ statistically from each other, whole means with different letters do differ (p≤0.05); n- number of participants and group 2: patients with paracoccidioidomycosis and without relapse. Statistical analysis: Student’s *t*-test.
(DOCX)

## Abbreviations

PCM: paracoccidioidomicose
IDD: Double immunodiffusion agar gel
ELISA: enzyme-linked immunosorbent assay
ESR: Erythrocyte sedimentation rate
CMX: cotrimoxazol
DTT: ditiotreitol
IAA: iodoacetamida
Ig: imunoglobulina
TF: transferrin
TFRC: transferrin receptor
HFE: h*emochromatosis*
EGF: epidermal growth factor
APOB: apolipoprotein B
RAB5A: member RAS oncogene family
ALB: serum albumin
TFR2: transferrin receptor 2
EGFR: epidermal growth factor receptor
CFTR: cystic fibrosis transmembrane conductance regulator (ATP-binding cassette sub-family C, member 7)
M6PR: mannose-6-phosphate receptor (cation dependent)
AF: acute / subacute clinical form
CF: chronic clinical form
HIF: hypoxia-inducible factors
CP: ceruloplasmin
IGFBP3: insulin-like growth fator binding protein
HIF1A: hypoxia inducible fator 1
SLC40A1: solute carrier faily 40 (iron-regulated transporter)
MIS12: MIND kinetochore complex component
HEPH: hephaestin
APOA1: apoliprotein A-I
HP: haptoglobin
HBB: hemoglobin, beta
CD163: CD163 molecule
HBA2: Hemoglobin, alpha 2
APOA1: apolipoprotein A-I
HBA1: hemoglobin, alpha 1
HPX: hemopexin
CRP: C-reactive protein
HPR: haptoglobin-related protein
CO: monóxido de carbono
