# Supplementary material for: Ceruloplasmin, transferrin and apolipoprotein A-II play important role in treatment's follow-up of paracoccidioidomycosis patients
Source: PLoS One. 2018 Oct 25;13(10):e0206051. doi: 10.1371/journal.pone.0206051 (PMC6201901; doi:10.1371/journal.pone.0206051)
Supplement: S1 Table — A-C. Serum protein quantification as spectral count, presented as mean and standard deviation in patients with paracoccidioidomycosis—G1 and G2 groups and healthy subjects—G3 group, before treatment (at admission). Means with the same letters in bold do not differ statistically from each other, whole means with different letters do differ (p≤0.05); AF—acute / subacute clinical form; CF- chronic clinical form; n- number of participants and … sequence not in database; Group 1: patients with paracoccidioidomycosis and relapse; Group 2: patients with paracoccidioidomycosis and without relapse; Group 3: healthy individuals. Statistical analysis: analysis of variance and Tukey test. (DOCX) [file pone.0206051.s001.docx]

**Table S1A.** Serum protein quantification as spectral count, presented as mean and standard deviation in patients with paracoccidioidomycosis - G1 and G2 groups and healthy subjects - G3 group, before treatment (at admission).

| **Protein** | **Access code** | **Molecular mass (kDa)** | **Coverage rate**  **(%)** | **[G1] *P. brasiliensis***  **with relapse**  **AF (n=1) / CF (n=2)** | **[G2] *P. brasiliensis***  **without relapse**  **AF (n=2) / CF (n=2)** | **[G3] Control group**  **(n=3)** | **Main function** | ***p*** |
| --- | --- | --- | --- | --- | --- | --- | --- | --- |
| **1.** *Serum albumin* | P02768.2 | 69 | 79 | 127.0 ± 48.9 | 140.1 ± 23.5 | 197.1 ± 16.1 | Transport | 0.06 |
| **2.** *Transferrin* | P02787.3 | 77 | 34 | 17.7 ± 5.5 **ab** | 15.2 ± 2.7 **b** | 26.6 ± 6.7 **a** | Transport | **0.04** |
| **3.** *Apoliprotein A-I* | P02647.1 | 31 | 36 | 9.11 ± 9.5 | 13.7 ± 3.5 | 23.5 ± 3.6 | Transport | 0.06 |
| **4.** *Haptoglobin* | P00738.1 | 45 | ... | 14.0 ± 1.4 **ab** | 24.9 ± 6.3 **a** | 12.5 ± 5.1 **b** | Immunomodulatory | **0.03** |
| **5.** *Ig kappa chain C region* | P01834.2 | … | … | 9.5 ± 4.5 | 18.9 ± 9.1 | 18.0 ± 1.6 | Immunomodulatory | 0.20 |
| **6.** *Ig gamma-1 chain C region* | P01857.1 | ... | ... | 9.6 ± 4.8 | 12.9 ± 5.6 | 8.2 ± 1.58 | Immunomodulatory | 0.42 |
| **7.** *Ig lambda-2 chain C region* | P0CG05.1 | ... | 75 | 8.0 ± 5.1 | 13.5 ± 6.6 | 9.7 ± 3.6 | Immunomodulatory | 0.43 |
| **8.** *Alpha-2-macroglobulin* | P01023.3 | 163 | 05 | 5.4 ± 3.6 | 8.3 ± 5.9 | 9.3 ± 4.4 | Activate/regulate the complement system | 0.63 |
| **9.** *Ig alpha-1 chain C region* | P01876.2 | 38 | 29 | 7.2 ± 2.1 | 11.2 ± 3.02 | 10.1 ± 2.0 | Immunomodulatory | 0.18 |
| **10.** *Alpha-1-antitrypsin* | P01009.3 | 47 | 08 | 3.3 ± 3.1 | 9.0 ± 2.2 | 5.1 ± 3.6 | Activate the coagulation pathway / protease-inhibition | 0.09 |
| **11.** *Hemopexin* | P02790.2 | 52 | 19 | 3.1 ± 4.0 | 5.5 ± 2.2 | 8.6 ± 3.0 | Transport | 0.15 |
| **12.** *Ig gamma-2 chain C region* | P01859.2 | 36 | 29 | 1.4 ± 1.2 | 4.5 ± 1.3 | 4.78 ± 2.5 | Immunomodulatory | 0.08 |

Means with the same letters in bold do not differ statistically from each other, whole means with different letters do differ (p≤0.05); AF - acute / subacute clinical form; CF- chronic clinical form; n- number of participants and … sequence not in database; Group 1: patients with paracoccidioidomycosis and relapse; Group 2: patients with paracoccidioidomycosis and without relapse; Group 3: healthy individuals. Statistical analysis: analysis of variance and Tukey test.

**Table S1B.** Serum protein quantification as spectral count, presented as mean and standard deviation in patients with paracoccidioidomycosis - G1 and G2 groups and healthy subjects - G3 group, before treatment (at admission).

| **Protein** | **Access code** | **Molecular mass (kDa)** | **Coverage rate**  **(%)** | **[G1] *P. brasiliensis***  **with relapse**  **AF (n=1) / CF (n=2)** | **[G2] *P. brasiliensis***  **without relapse**  **AF (n=2) / CF (n=2)** | **[G3] Control group**  **(n=3)** | **Main function** | ***p*** |
| --- | --- | --- | --- | --- | --- | --- | --- | --- |
| **13.** *Alpha-1-acid-glycoprotein* | P02763.1 | 24 | 19 | 2.6 ± 3.0 | 4.9 ± 2.1 | 3.6 ± 1.5 | Transport | 0.48 |
| **14.** *Complement C3* | P01024.2 | 187 | 05 | 1.1 ± 1.6 | 2.0 ± 1.2 | 4.7 ± 3.7 | Immunomodulatory | 0.20 |
| **15.** *Apolipoprotein A-II* | P02652.1 | 11 | 58 | 0.4 ± 0.5 **b** | 0.8 ± 0.5 **ab** | 4.8 ± 2.1 **a** | Transport / lipid metabolism | **0.01** |
| **16.** *Ig gamma-3 chain C region* | P01860.2 | ... | ... | 0.8 ± 1.2 | 0.8 ± 0.6 | 1.7 ± 1.0 | Immunomodulatory | 0.43 |
| **17.** *Ig gamma-4 chain C region* | P01861.1 | 36 | 23 | 2.8 ± 5.0 | 3.7 ± 5.1 | 0.8 ± 1.5 | Immunomodulatory | 0.70 |
| **18.** *Vitamin D-Binding Protein* | P02774.1 | 53 | 05 | 0.2 ± 0.3 | 0.7 ± 0.6 | 1.6 ± 0.5 | Immunomodulatory | 0.06 |
| **19.** *Ceruloplasmin* | P00450.1 | 122 | 01 | 0.0 ± 0.0 | - 1. ± 1.1 | 0.1 ± 0.1 | Transport | 0.10 |
| **20.** *Complement C4-A* | P0C0L4.2 | 193 | 01 | 0.2 ± 0.3 | 0.5 ± 0.9 | 1.0 ± 0.3 | Immunomodulatory | 0.42 |
| **21.** *Alpha-1-antichymotrypsin* | P01011.2 | 48 | 02 | 0.2 ± 0.3 **ab** | 0.6 ± 0.2 **a** | 0.0 ± 0.0 **b** | Protease-inhibition / lipid metabolism | **0.03** |
| **22.** *Kininogen* | P01042.2 | 72 | 02 | 0.1 ± 0.1 | 0.1 ± 0.1 | 0.2 ± 0.3 | Protease-inhibition | 0.88 |

Means with the same letters in bold do not differ statistically from each other, whole means with different letters do differ (p≤0.05); AF - acute / subacute clinical form; CF- chronic clinical form; n- number of participants and … sequence not in database; Group 1: patients with paracoccidioidomycosis and relapse; Group 2: patients with paracoccidioidomycosis and without relapse; Group 3: healthy individuals. Statistical analysis: analysis of variance and Tukey test.

**Table S1C.** Serum protein quantification as spectral count, presented as mean and standard deviation in patients with paracoccidioidomycosis - G1 and G2 groups and healthy subjects - G3 group, before treatment (at admission).

| **Protein** | **Access code** | **Molecular mass (kDa)** | **Coverage rate**  **(%)** | **[G1] *P. brasiliensis***  **with relapse**  **AF (n=1) / CF (n=2)** | **[G2] *P. brasiliensis***  **without relapse**  **AF (n=2) / CF (n=2)** | **[G3] Control group**  **(n=3)** | **Main function** | ***p*** |
| --- | --- | --- | --- | --- | --- | --- | --- | --- |
| **23.** *Ig alpha-2 chain C region* | P01877.3 | 37 | 08 | 0.0 ± 0.0 | 0.0 ± 0.0 | 0.0 ± 0.0 | Immunomodulatory | 1.00 |
| **24.** *Beta-globin* | P68871.2 | 16 | 09 | 0.3 ± 0.5 | 0.5 ± 1.0 | 0.3 ± 0.3 | Transpot | 0.94 |
| **25.** *Ig kappa chain V-III* | P04433.1 | 13 | 08 | 0.2 ± 0.3 | 0.7 ± 1.5 | 0.3 ± 0.5 | Immunomodulatory | 0.78 |
| **26.** *Beta-2-glycoprotein 1* | P02749.3 | 38 | 15 | 0.1 ± 0.1 | 0.3 ± 0.6 | 0.8 ± 0.1 | Matrix protein | 0.17 |
| **27.** *Ig heavy chain V-III TIL* | P01764.2 | 12 | 26 | 0.3 ± 0.5 | 1.4 ± 2.2 | 0.1 ± 0.1 | Immunomodulatory | 0.49 |
| **28.** *Complement factor B* | P00751.2 | 86 | 01 | 0.0 ± 0.0 | 0.4 ± 0.4 | 0.5 ± 0.5 | Immunomodulatory | 0.25 |
| **29.** *Alpha-globin* | P69905.2 | 15 | 11 | 0.0 ± 0.0 | 0.1 ± 0.1 | 0.0 ± 0.0 | Transport | 0.53 |

AF - acute / subacute clinical form; CF- chronic clinical form; n- number of participants and … sequence not in database; Group 1: patients with paracoccidioidomycosis and relapse; Group 2: patients with paracoccidioidomycosis and without relapse; Group 3: healthy individuals. Statistical analysis: analysis of variance and Tukey test.
