# Supplementary material for: Ceruloplasmin, transferrin and apolipoprotein A-II play important role in treatment's follow-up of paracoccidioidomycosis patients
Source: PLoS One. 2018 Oct 25;13(10):e0206051. doi: 10.1371/journal.pone.0206051 (PMC6201901; doi:10.1371/journal.pone.0206051)
Supplement: S2 Table — A-B. Serum protein quantification as spectral count, presented as mean and standard deviation in five patients with paracoccidioidomycosis and with disease relapse (Group G1), before treatment (at admission), at the time of relapse and after treatment of relapse. Means with the same letters in bold do not differ statistically from each other, whole means with different letters do differ (p≤0.05); n- number of participants and group 1: patients with paracoccidioidomycosis and relapse. Statistical analysis: analysis of variance and Tukey test. (DOCX) [file pone.0206051.s002.docx]

**Table S2A.** Serum protein quantification as spectral count, presented as mean and standard deviation in five patients with paracoccidioidomycosis and with disease relapse (Group G1), before treatment (at admission), at the time of relapse and after treatment of relapse.

| **Protein** | **Access code** | **Molecular mass (kDa)** | **Coverage rate**  **(%)** | **Before treatment (n=3)*** | **At relapse (n=5)** | **After the treatment of relapse (n=5)** | **Main function** | ***p*** |
| --- | --- | --- | --- | --- | --- | --- | --- | --- |
| **1.** *Serum albumin* | P02768.2 | 69 | 70 | 130.0 ± 49.6 | 189.9 ± 37.2 | 156.7 ± 10.7 | Transport | 0.08 |
| **2.** *Transferrin* | P02787.3 | 77 | 31 | 18.6 ± 6.1 **b** | 27.5 ± 4.0 **ab** | 29.5 ± 4.5 **a** | Transport | **0.02** |
| **3.** *Apoliprotein A-I* | P02647.1 | 31 | 38 | 9.8 ± 10.0 | 19.1 ± 2.0 | 17.8 ± 4.3 | Transport | 0.09 |
| **4.** *Haptoglobin* | P00738.1 | 45 | ... | 15.4 ± 0.7 | 14.0 ± 5.2 | 12.9 ± 4.5 | Immunomodulatory | 0.74 |
| **5.** *Ig kappa chain C region* | P01834.2 | … | … | 10.2 ± 4.9 | 11.4 ± 4.9 | 9.3 ± 4.9 | Immunomodulatory | 0.79 |
| **6.** *Ig gamma-1 chain C region* | P01857.1 | ... | ... | 10.4 ± 5.3 | 10.3 ± 3.4 | 10.3 ± 4.5 | Immunomodulatory | 0.99 |
| **7.** *Ig lambda-2 chain C region* | P0CG05.1 | ... | 81 | 9.0 ± 5.6 | 10.2 ± 1.2 | 9.3 ± 2.3 | Immunomodulatory | 0.84 |
| **8.** *Alpha-2-macroglobulin* | P01023.3 | 163 | 07 | 5.67 ± 3.67 | 15.33 ± 7.04 | 8.47 ± 5.14 | Activate/regulate the complement system | 0.22 |
| **9.** *Ig alpha-1 chain C region* | P01876.2 | 38 | 15 | 7.4 ± 2.0 | 8.7 ± 5.1 | 6.3 ± 4.2 | Immunomodulatory | 0.70 |
| **10.** *Alpha-1-antitrypsin* | P01009.3 | 47 | 07 | 3.4 ± 3.3 | 5.3 ± 2.2 | 3.8 ± 1.2 | Activate the coagulation pathway / protease-inhibition | 0.43 |
| **11.** *Hemopexin* | P02790.2 | 52 | 10 | 3.1 ± 4.0 | 6.3 ± 2.7 | 5.8 ± 3.2 | Transport | 0.40 |
| **12.** *Ig gamma-2 chain C region* | P01859.2 | 36 | 16 | 1.7 ± 1.5 | 2.5 ± 1.1 | 1.5 ± 0.9 | Immunomodulatory | 0.42 |

Means with the same letters in bold do not differ statistically from each other, whole means with different letters do differ (p≤0.05); n- number of participants and group 1: patients with paracoccidioidomycosis and relapse. Statistical analysis: analysis of variance and Tukey test.

**Table S2B.** Serum protein quantification as spectral count, presented as mean and standard deviation in five patients with paracoccidioidomycosis and with disease relapse (Group G1), before treatment (at admission), at the time of relapse and after treatment of relapse.

| **Protein** | **Access code** | **Molecular mass (kDa)** | **Coverage rate**  **(%)** | **Before treatment (n=3)*** | **At relapse (n=5)** | **After the treatment of relapse (n=5)** | **Main function** | ***p*** |
| --- | --- | --- | --- | --- | --- | --- | --- | --- |
| **13.** *Alpha-1-acid-glycoprotein* | P02763.1 | 24 | 04 | 2.7 ± 3.1 | 1.0 ± 0.9 | 1.6 ± 1.2 | Transport | 0.43 |
| **14.** *Complement C3* | P01024.2 | 187 | 01 | 1.3 ± 2.0 | 2.0 ± 1.4 | 1.1 ± 1.2 | Immunomodulatory | 0.60 |
| **15.** *Apolipoprotein A-II* | P02652.1 | 11 | 10 | 0.44 ± 0.5 **b** | 2.07 ± 0.7 **a** | 1.73 ± 0.4 **ab** | Transport / lipid metabolism | **0.01** |
| **16.** *Ig gamma-3 chain C region* | P01860.2 | ... | ... | 1.1 ± 1.6 | 1.7 ± 1.3 | 1.7 ± 1.8 | Immunomodulatory | 0.86 |
| **17.** *Ig gamma-4 chain C region* | P01861.1 | 36 | 16 | 3.8 ± 6.5 | 0.3 ± 0.4 | 0.0 ± 0.0 | Immunomodulatory | 0.22 |
| **18.** *Vitamin D-Binding Protein* | P02774.1 | 53 | 04 | 0.2 ± 0.4 | 0.7 ± 0.8 | 1.0 ± 7.0 | Immunomodulatory | 0.36 |
| **19.** *Ceruloplasmin* | P00450.1 | 122 | 01 | 0.0 ± 0.0 | 0.5 ± 0.9 | 0.0 ± 0.0 | Transport | 0.28 |
| **20.** *Complement C4-A* | P0C0L4.2 | 193 | 01 | 0.2 ± 0.4 | 0.5 ± 0.9 | 0.1 ± 0.3 | Immunomodulatory | 0.58 |
| **21.** *Alpha-1-antichymotrypsin* | P01011.2 | 48 | 07 | 0.2 ± 0.4 | 0.4 ± 0.6 | 0.0 ± 0.0 | Protease-inhibition / lipid metabolism | 0.35 |
| **22.** *Kininogen* | P01042.2 | 72 | ... | 0.11 ± 0.19 | 0.27 ± 0.37 | 0.20 ± 0.18 | Protease-inhibition | 0.74 |

Means with the same letters in bold do not differ statistically from each other, whole means with different letters do differ (p≤0.05); n- number of participants and group 1: patients with paracoccidioidomycosis and relapse. Statistical analysis: analysis of variance and Tukey test.
