# Supplementary material for: Ceruloplasmin, transferrin and apolipoprotein A-II play important role in treatment's follow-up of paracoccidioidomycosis patients
Source: PLoS One. 2018 Oct 25;13(10):e0206051. doi: 10.1371/journal.pone.0206051 (PMC6201901; doi:10.1371/journal.pone.0206051)
Supplement: S3 Table — A-B. Serum protein quantification as spectral count, presented as mean and standard deviation in four patients with paracoccidioidomycosis (Group G2) before and after treatment. Means with the same letters in bold do not differ statistically from each other, whole means with different letters do differ (p≤0.05); n- number of participants and group 2: patients with paracoccidioidomycosis and without relapse. Statistical analysis: Student’s t-test. (DOCX) [file pone.0206051.s003.docx]

**Table S3A.** Serum protein quantification as spectral count, presented as mean and standard deviation in four patients with paracoccidioidomycosis (Group G2) before and after treatment.

| **Protein** | **Access code** | **Molecular mass (kDa)** | **Coverage rate**  **(%)** | **Before treatment (n=4)** | **After treatment (n=4)** | **Main function** | ***p*** |
| --- | --- | --- | --- | --- | --- | --- | --- |
| **1.** *Serum albumin* | P02768.2 | 69 | 57 | 142.6 ± 23.8 | 133.3 ± 22.9 | Transport | 0.68 |
| **2.** *Transferrin* | P02787.3 | 77 | 14 | 16.0 ± 2.6 | 20.2 ± 8.5 | Transport | 0.43 |
| **3.** *Apoliprotein A-I* | P02647.1 | 31 | 10 | 14.3 ± 3.5 | 10.9 ± 6.3 | Transport | 0.49 |
| **4.** *Haptoglobin* | P00738.1 | 45 | 10 | 25.6 ± 6.3 **a** | 10.9 ± 10.3 **b** | Immunomodulatory | **0.03** |
| **5.** *Ig kappa chain C region* | P01834.2 | … | … | 20.3 ± 9.6 | 9.2 ± 6.3 | Immunomodulatory | 0.12 |
| **6.** *Ig gamma-1 chain C region* | P01857.1 | ... | ... | 13.3 ± 5.8 | 9.9 ± 1.1 | Immunomodulatory | 0.27 |
| **7.** *Ig lambda-2 chain C region* | P0CG05.1 | ... | 28 | 14.6 ± 6.6 | 8.4 ± 4.0 | Immunomodulatory | 0.12 |
| **8.** *Alpha-2-macroglobulin* | P01023.3 | 163 | 02 | 8.83 ± 5.97 | 3.67 ± 1.41 | Activate/regulate the complement system | 0.25 |
| **9.** *Ig alpha-1 chain C region* | P01876.2 | 38 | 06 | 11.6 ± 3.1 | 7.3 ± 2.0 | Immunomodulatory | 0.15 |
| **10.** *Alpha-1-antitrypsin* | P01009.3 | 47 | 04 | 9.7 ± 1.9 | 3.5 ± 2.9 | Activate the coagulation pathway / protease-inhibition | 0.07 |
| **11.** *Hemopexin* | P02790.2 | 52 | 05 | 5.8 ± 2.2 | 3.3 ± 4.7 | Transport | 0.51 |
| **12.** *Ig gamma-2 chain C region* | P01859.2 | 36 | 11 | 5.4 ± 1.4 **a** | 1.7 ± 1.8 **b** | Immunomodulatory | **0.04** |
| **13.** *Alpha-1-acid-glycoprotein* | P02763.1 | 24 | 17 | 4.9 ± 2.2 **a** | 1.8 ± 2.7 **b** | Transport | **0.04** |
| **14.** *Complement C3* | P01024.2 | 187 | 03 | 2.42 ± 1.5 | 1.08 ± 2.17 | Immunomodulatory | 0.24 |

Means with the same letters in bold do not differ statistically from each other, whole means with different letters do differ (p≤0.05); n- number of participants and group 2: patients with paracoccidioidomycosis and without relapse. Statistical analysis: Student’s *t*-test.

**Table S3B.** Serum protein quantification as spectral count, presented as mean and standard deviation in four patients with paracoccidioidomycosis (Group G2) before and after treatment.

| **Protein** | **Access code** | **Molecular mass (kDa)** | **Coverage rate**  **(%)** | **Before treatment (n=4)** | **After treatment (n=4)** | **Main function** | ***p*** |
| --- | --- | --- | --- | --- | --- | --- | --- |
| **15.** *Apolipoprotein A-II* | P02652.1 | 11 | 10 | 0.83 ± 0.58 | 1.67 ± 0.72 | Transport / lipid metabolism | 0.24 |
| **16.** *Ig gamma-3 chain C region* | P01860.2 | ... | ... | 0.9 ± 0.6 | 0.6 ± 1.2 | Immunomodulatory | 0.57 |
| **17.** *Ig gamma-4 chain C region* | P01861.1 | 36 | 11 | 4.0 ± 5.2 | 0.2 ± 0.3 | Immunomodulatory | 0.25 |
| **18.** *Vitamin D-Binding Protein* | P02774.1 | 53 | 08 | 0,75 ± 0.69 | 0.0 ± 0.0 | Immunomodulatory | 0.12 |
| **19.** *Ceruloplasmin* | P00450.1 | 122 | 03 | 1.33 ± 1.25 | 0.0 ± 0.0 | Transport | 0.12 |
| **20.** *Complement C4-A* | P0C0L4.2 | 193 | 01 | 0.58 ± 0.96 | 0.0 ± 0.0 | Immunomodulatory | 0.31 |
| **21.** *Alpha-1-antichymotrypsin* | P01011.2 | 48 | 04 | 0,75 ± 0.42 **a** | 0.0 ± 0.0 **b** | Protease-inhibition / lipid metabolism | **0.04** |
| **22.** *Kininogen* | P01042.2 | 72 | ... | 0.17 ± 0.19 | 0.17 ± 0.33 | Protease-inhibition | 1.00 |

Means with the same letters in bold do not differ statistically from each other, whole means with different letters do differ (p≤0.05); n- number of participants and group 2: patients with paracoccidioidomycosis and without relapse. Statistical analysis: Student’s *t*-test.
